# Smartphone image dataset for machine learning-based monitoring and analysis of mango growth stages

**DOI:** 10.1016/j.dib.2025.111780

**Published:** 2025-06-26

**Authors:** Sayem Kabir, Md Fokrul Islam Akon, Mohammad Rifat Ahmmad Rashid, Maheen Islam, Taskeed Jabid, Mohammad Manzurul Islam, Md Sawkat Ali

**Affiliations:** Department of Computer Science and Engineering, East West University, Dhaka 1212, Bangladesh

**Keywords:** Mango growth stage, Agricultural dataset, Data preprocessing, Machine learning, Public dataset

## Abstract

Machine learning and artificial intelligence have gained widespread popularity across various sectors in Bangladesh, with the notable exception of the agriculture industry. While wealthier nations have extensively adopted machine learning and deep learning techniques in agriculture, Bangladesh's agricultural sector has been slower to follow suit. A key factor in the success of any machine learning model is the availability of high-quality datasets. However, practitioners in Bangladesh's mango industry face challenges in leveraging these advanced computational methods due to the lack of standardized and publicly accessible datasets. A well-structured dataset is essential for developing accurate models and reducing misclassification in real-world applications. To address this gap, we have developed a standardized image dataset capturing different stages of mango growth. The dataset, collected between April and June at an orchard on the East West University campus in Bangladesh, consists of 2004 images, each annotated and categorized into four distinct growth stages: early-fruit, premature, mature, and ripe. Although the dataset was created using mangoes from Bangladesh, the growth stages documented are representative of mango development globally, making this dataset applicable to mango cultivation in other countries. The dataset is organized into four folders, each containing both images and corresponding annotation files. We anticipate that this dataset will serve as a valuable resource for researchers and practitioners working in the field of automated agriculture, facilitating the development of machine learning models for monitoring and analyzing mango growth stages.

Specifications Table

**Table:** 

Subject	Agriculture Engineering, Machine Learning, Computer Vision and Data Science.
Specific subject area	Machine learning based classification of mango growth stages
Data format	RAW: JPEG Conversion: jpg Annotation: XML and json
Type of data	Images of size 640×640 pixels having RGB color in jpg format
Data collection	We captured images of mangoes at different stages of growth using three distinct smartphones. Each image was originally taken at a resolution of approximately 4640×3472 pixels, and the images were saved in JPEG format. These samples were collected from a mango orchard at East West University in Bangladesh over the course of regular days from April to June, with images taken every 3 to 4 days. The dataset was categorized into four groups: early-fruit, premature, mature, and ripe. In total, we captured 1057 raw images, with the ripe mangoes photographed against both white and brown backgrounds. This precaution was necessary because, as mangoes ripen and mature, they are typically removed from the trees to protect them from damage caused by birds and insects. After the initial image collection, the dataset was processed by resizing the images and filtering out low-quality ones. Various augmentation techniques, including zooming, rotation, and flipping, were applied, resulting in a final dataset of 2004 images. Each category contains approximately 500 images, providing a balanced dataset for machine learning tasks.
Data source location	Institute: East West University City: Aftabnagar, Dhaka Country: Bangladesh Latitude: 23.768820183974263, Longitude: 90.42552373253056
Data accessibility	Repository name: Mendeley Data Data identification number: 10.17632/5snwpzdtzs.1 Direct URL to data: https://data.mendeley.com/datasets/5snwpzdtzs/1
Related research article	None

## 1 Value of the Data

• We have created a detailed image dataset capturing various stages of mango growth in Bangladesh, one of the largest mango-producing countries. The initial dataset consists of 1057 manually captured images from mango orchards at East West University. The dataset consists of 2004 high-quality images, enhanced with augmentation techniques such as rotation, flipping, and zooming.
• This dataset serves as a valuable resource for researchers in computer vision and precision agriculture. It enables the development of advanced machine learning models for mango growth classification, yield prediction, and quality assessment [1].
• We applied various data validation techniques, including resizing, zooming, flipping, and manual annotation. These steps improve dataset consistency and accuracy, ensuring robust and reliable model performance [2].
• The dataset is publicly available and formatted for seamless integration into machine learning frameworks. This reduces research time and preprocessing effort, accelerating innovation in AI-driven agricultural solutions.
• The dataset is suitable for transfer learning, allowing adaptation to mango cultivation in other tropical regions. It supports cross-regional applications in automated harvesting, supply chain optimization, and disease monitoring [3].

## 2 Background

**Fig. 1:**
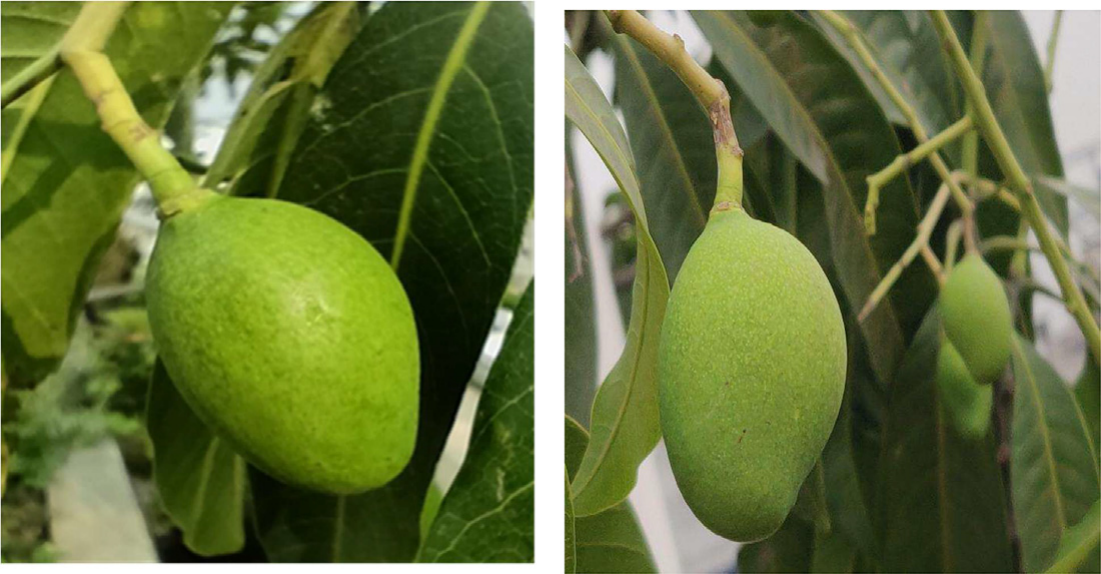
Mango early-fruit growth stage.

**Fig. 2:**
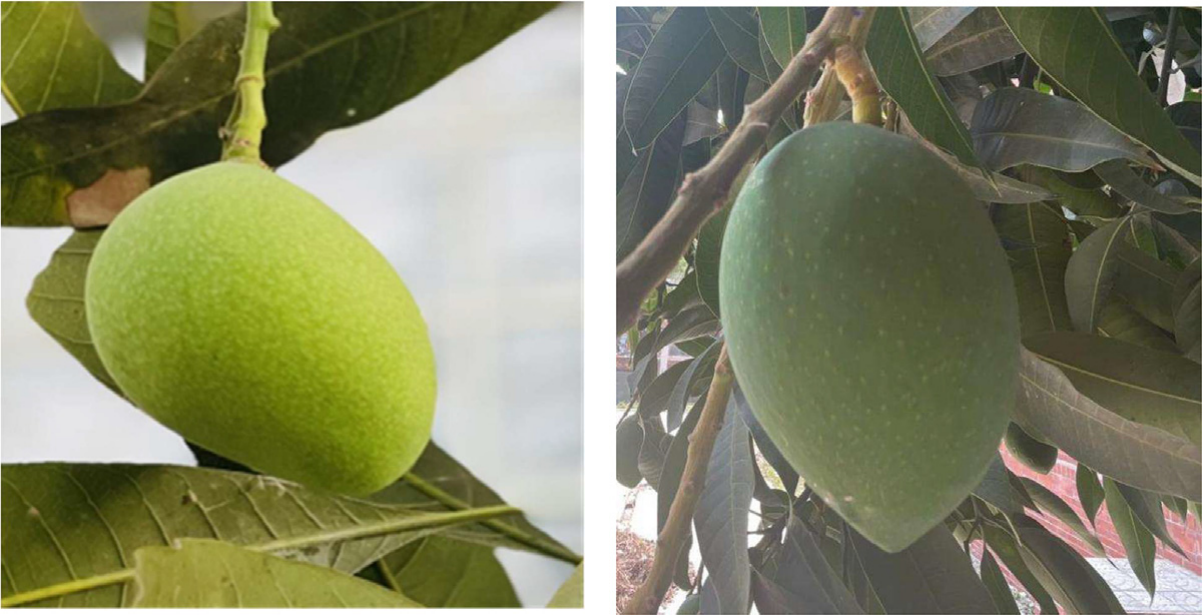
Mango premature growth stage.

**Fig. 3:**
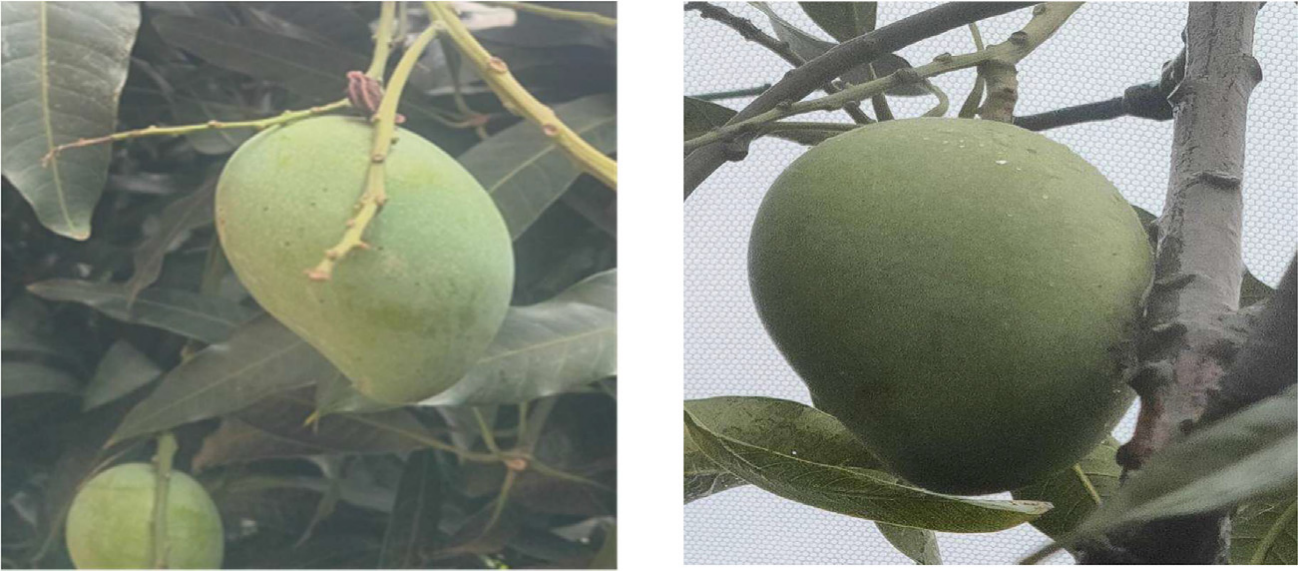
Mango mature growth stage.

**Fig. 4:**
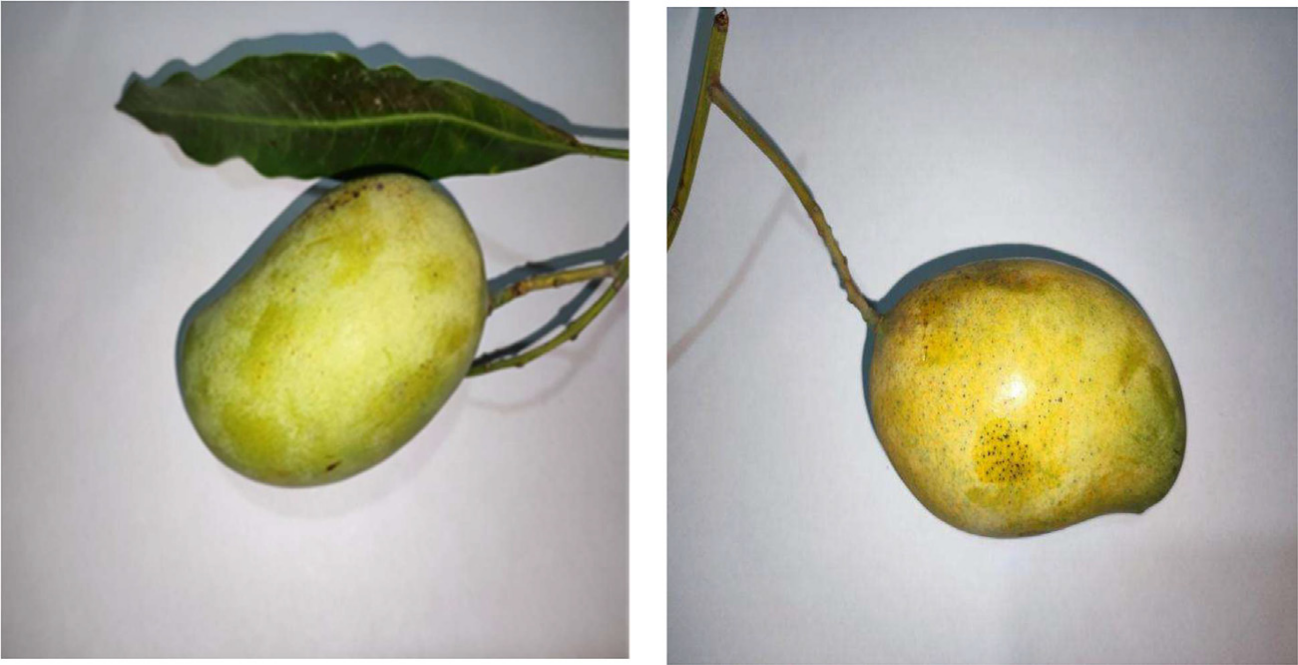
Mango ripe growth stage.

Mangoes (Mangifera indica), one of the most important tropical fruits in the world, are predominantly cultivated in Southern Asia, including regions such as Eastern India, China, Bangladesh, Myanmar, the Andaman Islands, and parts of Central America. In Bangladesh, the history of mango cultivation spans over 4000 years [[4], [5], [6], [7], [8]]. As the leading fruit crop in Asia and one of the top five fruits produced globally each year, mangoes belong to the Anacardiaceae family, with an annual yield of around 50 million metric tons [5]. Mango cultivation plays a crucial role in Bangladesh’s agricultural economy, particularly in major mango-producing regions like Rajshahi, Chapai Nawabganj, and Satkhira, where it contributes significantly to both local markets and international exports. Advancements in computer vision, deep learning, and machine learning have enabled precise fruit growth monitoring and classification, but these methods heavily depend on high-quality, standardized datasets [[9], [10], [11]]. While AI has been widely adopted in agriculture in developed countries, mango growth classification research remains limited in Bangladesh and similar tropical regions due to a lack of publicly available, well-annotated datasets. Existing datasets often suffer from class imbalance, poor annotation standards, and limited geographic diversity, making them unsuitable for scalable AI-driven agricultural solutions [[12], [13], [14]]. To address this gap, our study introduces a comprehensive, publicly accessible dataset capturing four distinct growth stages of mangoes in real-world orchard conditions. The dataset is designed to support machine learning-based classification, real-time monitoring, and decision-making for mango cultivation. This dataset sets a new benchmark for mango growth classification, enhancing the accuracy, robustness, and scalability of AI applications in precision agriculture (Fig. 1, Fig. 2, Fig. 3, Fig. 4, Table 1)

**Table 1:** Description of data collection.

Fruit	Mango
Growing Period	3 Months
Sample taking time	April 4 - June 30 Shooting every 3-4 days The times are all daytime hours
Location	Latitude: 23.768820183974263, Longitude: 90.42552373253056
Climate	Sunny Day
Temperature	32-38° Approximately

## 3 Data Description

The dataset used in this research consists of 2004 images of mangoes at various stages of growth, categorized into four groups: early-fruit, premature, mature, and ripe. Researchers can leverage this dataset to explore and test different computer vision and image processing techniques for identifying and detecting these growth stages. All images were manually captured using a smartphone camera on the East West University campus in Bangladesh. The original resolution of the photos, which was 4640×3472 pixels, was uniformly resized to 640×640 pixels to ensure consistency across the dataset. Details regarding the data collection process are provided in Table 2.

**Table 2:** Description of the dataset for the growth stage of mango.

Class	Early-fruit	Premature	Mature	Ripe
Number of images	468	526	514	496

We used the open-source annotation program Roboflow to methodically annotate the mangoes in the 2004 pictures in our collection. As shown in Table 3, the comments divided the mangoes into four different categories: early-fruit, premature, mature, and ripe.

**Table 3:** Brief description of dataset folder.

Fruit	Mango
Number of growth stage	4
Annotation file format	XML, JSON
Dataset size	Size of each image: 25 – 160 KB Folder size of images (COCO): 76.8 MB Folder size of images (VOC): 84.1 MB Annotation folder size (COCO): 685 KB Annotation folder size (VOC): 7.85 MB Dataset folder size: 160 MB

### 3.1 Description of the mango growth

#### 3.1.1 Early-fruit

In mango trees, the term “early fruit” refers to the first fruit that begins to develop after successful pollination. This stage is crucial because it sets the foundation for the fruit's overall quality and growth. Understanding the characteristics and needs of early-stage mango fruit is important for ensuring optimal development and final yield. When the mango fruit initially forms from fertilized blossoms, it is usually small and green. At this point, the fruit is actively growing in both size and internal structure, and the seed inside also begins to form. However, early fruits are generally more susceptible to diseases, pests, and environmental stressors, which can negatively impact their growth and quality. Recognizing these vulnerabilities and addressing them early on can help improve the fruit’s health and the eventual yield.

#### 3.1.2 Premature

The premature growth stage of mango development is a crucial period during which the fruit undergoes substantial growth but does not yet reach full maturity due to various physiological and environmental factors. Typically occurring about 20 days after the initial fruit set, this stage represents a transitional phase in the mango’s life cycle. Mangoes in this stage are characterized by their smaller size, greater firmness, and predominantly green color compared to fully mature fruits. Additionally, the organoleptic properties of mangoes at this stage differ significantly from those of ripe fruit. The immature mangoes lack the sweetness, juiciness, and complex flavor profile of fully ripened mangoes, which greatly impacts their taste and market value. Understanding the growth processes during this stage is essential for optimizing agricultural practices aimed at improving both fruit quality and yield.

#### 3.1.3 Mature

Mangoes reach their mature growth stage when they have completed their development. At this point, depending on the variety, mangoes will have achieved their ideal size, color, and texture. A key indicator of maturity is the change in color from green to yellow, signaling that the fruit is ready for consumption. The flesh of a mature mango develops a balanced sweet and slightly tangy flavor, accompanied by a pleasant aroma and smooth texture. To reach maturity, a perfect balance of environmental factors, such as temperature, sunlight, and water availability throughout the growing season, is essential. The transition from the premature stage to full maturity typically takes around eighteen days, during which the fruit undergoes significant internal changes.

#### 3.1.4 Ripe

The stage of ripe mango growth denotes the culmination of the fruit's growth and its ideal ripeness for ingestion. In order to protect them from birds and insects, mangoes need to be taken down from the tree as soon as possible and kept carefully. Mangoes have reached full maturity at this point, and depending on the type, they exhibit vivid colors ranging from green to yellow. The flesh of the fruit develops into a smooth, juicy, fragrant texture with a deep sweetness that characterizes its desired flavor profile. Studies reveal that this change from maturity to ripeness usually takes around 15 days, emphasizing how crucial timing is to obtaining mangoes at their most flavorful and nutritious.

### 3.2 Description of dataset folder

**Fig. 5:**
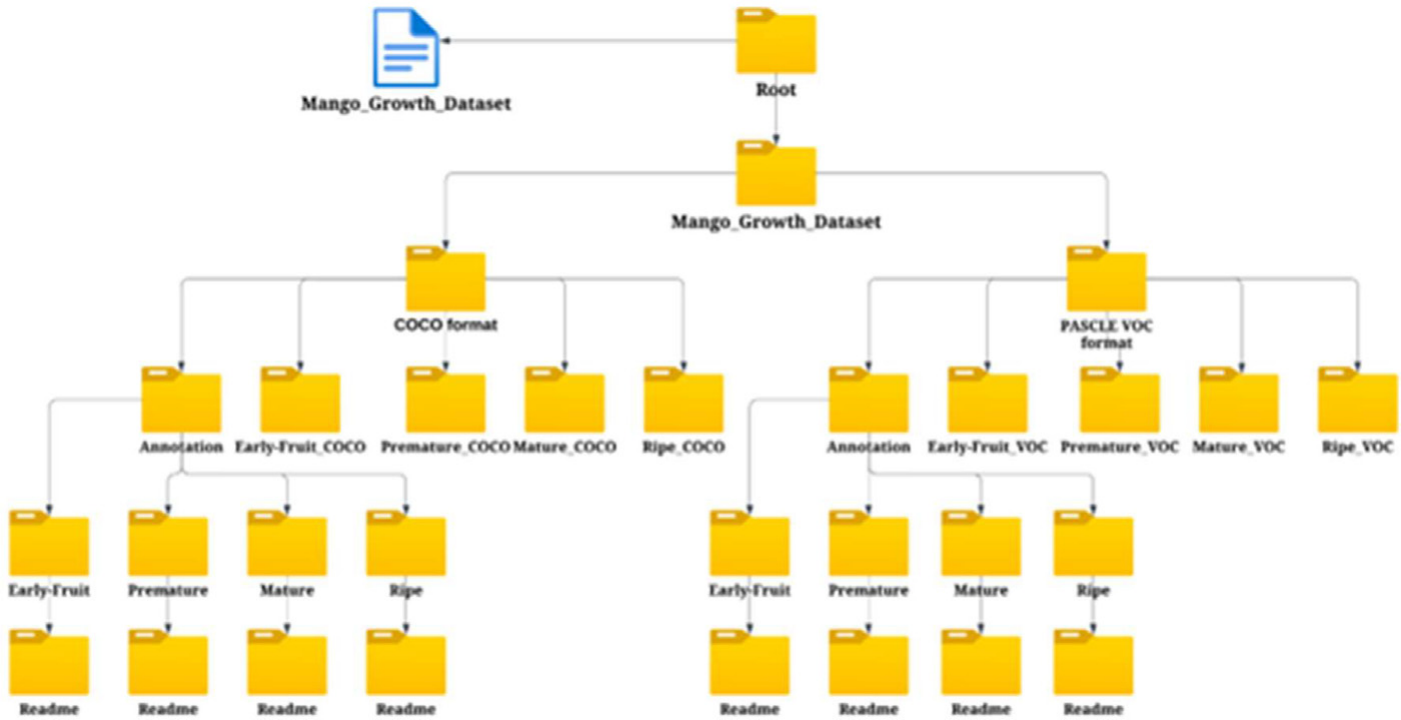
Folder structure.

2004 mango images were used to create the dataset's initial version. These images were divided into groups based on every one of mango growth stages, including early-fruit, premature, mature, and ripe. Table 4 presents a thorough description of the dataset, and Fig. 5 shows the folder organization.

**Table 4:** Description of camera device.

Manufacturers	Xiaomi, Samsung
Models	Xiaomi 11 Lite 5G NE Samsung Galaxy A04
Lens	Xiaomi camera f/1.8, 26 mm (wide) Samsung camera 50 MP, f/1.8, (wide)
Aperture	f/1.8
Camera Flash	Yes
Images	Size: 4640×3472 pixels Resulation: 96dpi Bit depth: 24

We use two different methods of storage for the Mango Growth Dataset for user convenience. To begin with, users can simply explore and inspect the dataset online by going to the Mango_Growth_Dataset folder. Second, downloading the Mango_Growth_Dataset.rar file is a simple process. To easily acquire the entire dataset, users can download and unzip this compressed file.

## 4 Experimental Design, Materials and Methods

The significance of data in machine learning cannot be overstated. Many experts believe that the success of modern machine learning systems is heavily dependent on the quality and volume of data available [15]. often more so than the choice of mathematical models. This perspective, known as the data-centric approach, contrasts with the traditional model-centric view. As a result, researchers must adhere to best practices when preparing datasets. These practices include selecting representative samples from real-world data, cleaning and enhancing the data, and ensuring accurate labeling [16]. In this section, we outline the steps we followed during our dataset preparation:

1. **Conduct comprehensive research on machine learning techniques and mango farming**: This step involves understanding the biological characteristics of mangoes and exploring machine learning methods applicable to image analysis in agricultural settings.
2. **Capture images of mangoes at different growth stages in a systematic manner**: To ensure temporal diversity and dataset comprehensiveness, images of mango trees were taken during four distinct growth cycles at prearranged intervals.
3. **Select mango orchards in consultation with horticultural experts**: We collaborated with specialists from East West University to choose the appropriate orchards for data collection.
4. **Preprocess and validate the collected dataset images**: This process involved converting images to a standard format, eliminating background noise, ensuring consistency in image size, and refining the data with the help of human experts. The images were then categorized and labeled to create a robust dataset for machine learning analysis [17].

**Fig. 6:**
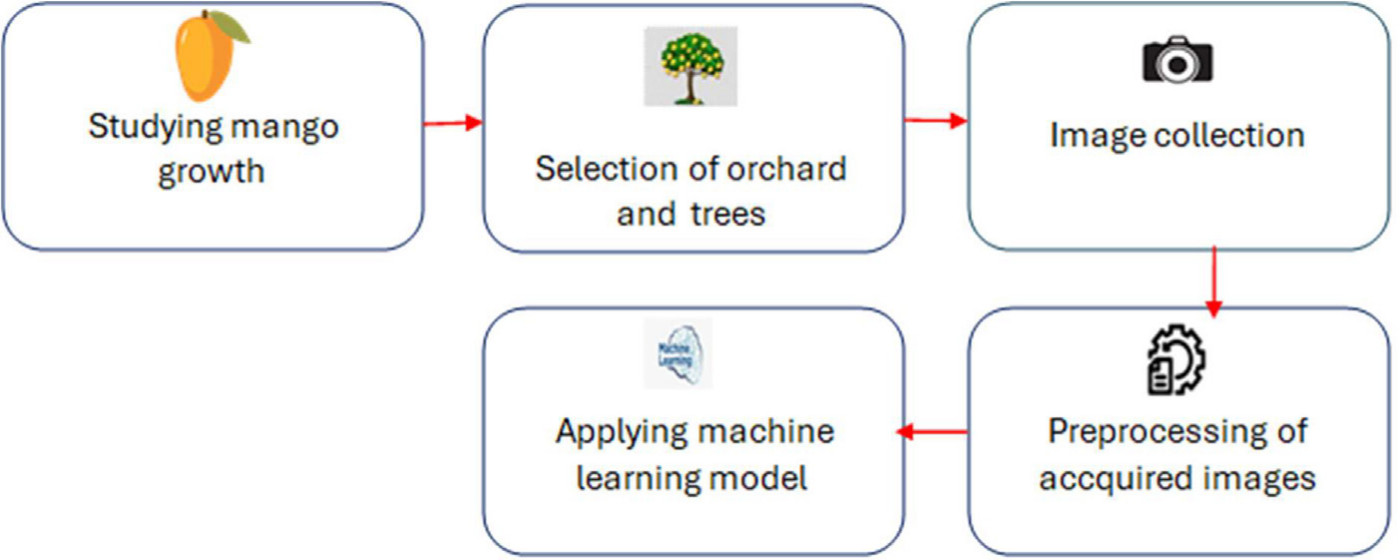
Flowchart of data preparation.

Each of these steps will be explained in detail below. The flowchart outlining the data preparation process is shown in Fig. 6.

### 4.1 Data collection

The study was conducted at a mango orchard located on the East West University campus in Aftabnagar, Dhaka, Bangladesh. The data collection period extended from April 2024 to June 2024. During this time, all mango fruits, except those considered ripe, were allowed to grow naturally. To capture the dataset, two smartphones were used: a Xiaomi with a 64-megapixel sensor and a Samsung with a 50-megapixel sensor. Both cameras feature f/1.8 aperture lenses (0.7 µm pixel size), CMOS sensors, autofocus, and optical image stabilization. The images were taken from a height of 5.7 feet, with the camera positioned between 2 to 8 meters away from the fruit. The photos were captured using the standard 1x zoom setting. A more detailed description of the image-capturing methodology is provided in Table 4.

### 4.2 Preprocessing

Initially, we converted 1057 mango images from JPEG to JPG format while preserving their original quality, using Roboflow, an online data labeling tool. To ensure uniformity and improve presentation, all images were resized to 640 × 640 pixels, as machine learning algorithms often require consistent image dimensions. The resized images were stored in the JPG format. During preprocessing, manual cleaning was conducted to remove any noise, such as scratches, present on the images. We also discarded extremely blurry, duplicate, or otherwise noisy images, resulting in a final set of approximately 1057 images. To enhance the dataset further, we applied image augmentation techniques such as rotation and zooming to specific images, increasing the total number of images to 2004 and adding more variability to the dataset. This process ensured that each of the four categories—early-fruit, premature, mature, and ripe—contained around 500 images.

We then annotated the mango dataset using the Roboflow platform. Each image was manually annotated by drawing bounding boxes around the mangoes and labeling them as “early-fruit,” “premature,” “mature,” or “ripe.” The annotation files were saved in both XML and COCO formats for compatibility with various machine learning frameworks. While we had thoroughly studied the mango growth cycle, we also sought the expertise of agricultural specialists to periodically verify the accuracy and reliability of our annotations. This collaborative approach helped improve the overall quality and dependability of the dataset.

## Limitations

The process of creating a machine learning dataset from scratch is far from simple; it demands a significant investment of time, effort, and human resources. However, the results of this labor are well worth it. A meticulously curated dataset, once made publicly available, becomes a valuable resource for thousands of machine learning researchers and practitioners. Below, we outline the main challenges we encountered during the preparation of our dataset:

• **Diversity of Mango Varieties**: Bangladesh is home to a wide range of mango varieties, making it difficult to select a single representative variety for the dataset.
• **Geographic Distribution of Mango Orchards**: To ensure a representative sample, researchers needed to carefully select orchards and plants. This was essential for capturing regional variations in mango growth and flavor. By including mangoes from diverse orchards, we were able to account for the environmental factors that influence mango development, ultimately creating a more reliable and widely applicable dataset.
• **Challenges in Capturing Images**: The height of mango trees presented significant challenges in photographing the fruit up close. Special techniques and equipment were required to overcome this issue and obtain high-quality images.

## Ethics Statement

The proposed data does not involve human subjects, animal experiments, or data collected from social media platforms.

## CRediT Author Statement

**Sayem Kabir:** Conceptualization, Methodology, Software, Writing original draft, Investigation, Data curation. **Md Fokrul Isalm Akon:** Conceptualization, Data curation. **Mohammad Rifat Ahmmad Rashid:** Validation, Writing – review & editing. **Maheen Islam**: Conceptualization, Data curation. **Taskeed Jabid**: Conceptualization, Data curation. **Mohammad Manzurul Islam**: Conceptualization, Data curation. **Md Sawkat Ali:** Conceptualization, Methodology, Supervision, Visualization, Project administration, Validation.

## Data Availability

Mendeley DataImage Dataset for Mango Growth Stages Analysis (Original data).
